# Cells and material-based strategies for regenerative endodontics

**DOI:** 10.1016/j.bioactmat.2021.11.015

**Published:** 2021-11-30

**Authors:** Zain Siddiqui, Amanda M. Acevedo-Jake, Alexandra Griffith, Nurten Kadincesme, Kinga Dabek, Dana Hindi, Ka Kyung Kim, Yoshifumi Kobayashi, Emi Shimizu, Vivek Kumar

**Affiliations:** a Department of Biomedical Engineering, New Jersey Institute of Technology, Newark, NJ, 07102, USA; b Department of Oral Biology, Rutgers School of Dental Medicine, Newark, NJ, 07103, USA; c Department of Endodontics, Rutgers School of Dental Medicine, Newark, NJ, 07103, USA; d Department of Chemicals and Materials Engineering, New Jersey Institute of Technology, Newark, NJ, 07102, USA; e Department of Biology, New Jersey Institute of Technology, Newark, NJ, 07102, USA

**Keywords:** Regenerative endodontics, Pulp regeneration, Tissue engineering, Stem cells, Scaffolds

## Abstract

<p class = "Abstract" style = "margin: 0 cm; line-height: 32px; font-size: 12 pt; font-family: "Times New Roman", serif; color: rgb(0, 0, 0); "><span lang = "EN-US">The carious process leads to inflammation of pulp tissue. Current care options include root canal treatment or apexification. These procedures, however, result in the loss of tooth vitality, sensitivity, and healing. Pulp capping and dental pulp regeneration are continually evolving techniques to regenerate pulp tissue, avoiding necrosis and loss of vitality. Many studies have successfully employed stem/progenitor cell populations, revascularization approaches, scaffolds or material-based strategies for pulp regeneration. Here we outline advantages and disadvantages of different methods and techniques which are currently being used in the field of regenerative endodontics. We also summarize recent findings on efficacious peptide-based materials which target the dental niche.<o:p></o:p></span></p>

## Highlights

• Pulp infection necessitates removal of necrotic, inflamed and infected tissue.
• Materials used clinically are inert (such as gutta percha, mineral trioxide aggregate).
• Recent developments in materials (angiogenic hydrogels, stem cell composites) have tuneable bioactivity.
• Dental pulp regeneration may now be possible through the use of bioactive systems, that guide regeneration.

## Graphical abstract

**Figure:**
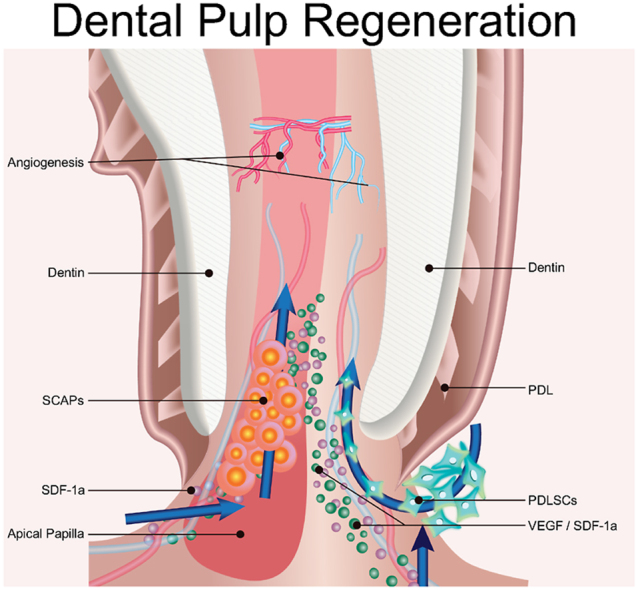

## Abbreviations

Human dental pulp stem cells: DPSCs
basic fibroblastic growth factor: bFGF
bone morphogenic protein: BMP
vascular endothelial growth factor: VEGF
platelet‐derived growth factor: PDGF
nerve growth factor: NGF
pathogen recognition receptors: PRRs
pathogen-associated molecular patterns: PAMPs
primary apical periodontitis: PAP
secondary apical periodontitis: SAP
calcium hydroxide: CH
mineral trioxide aggregate: MTA
Vital pulp therapy: VPT
dentin extracellular matrix: dECM
transforming growth factor-beta 1: TGFβ-1
insulin like growth factors: IGF
mesenchymal stem cells: MSCs
stem cells from human exfoliated deciduous teeth: SHED
stem cells from the apical part of the dental papilla: SCAP
periodontal ligament stem cells: PDLSC
dental follicle stem cells: DFSC
dental epithelial stem cells: DESCs
bone marrow-derived mesenchymal stem cells: BMMSCs
adipose-derived stem cells: ADSCs
embryonic stem cells: ESCs
induced pluripotent stem cells: iPSCs
hydroxyapatite: HA
silk fibroin: SF
ultra-small super paramagnetic iron oxide: USPIO
tricalcium phosphate: TCP
bone marrow stromal cells: BMSCs
poly-D,l-lactide and glycolide: PLG
porcine deciduous pulp stem/progenitor cells: pDPSCs
beta-tricalcium phosphate: b-TCP
stromal derived factor-1: SDF-1
induced mesenchymal stem cells: iMSCs
normal human epidermal keratinocytes: NHEKs
good manufacturing practice: cGMP
growth factors: GFs
electric pulp testing: EPT
Laser Doppler Flowmetry: LDF
American Dental Association: ADA
apical revascularization: AR
platelet-rich plasma: PRP
platelet rich fibrin: PRF
autologous ddoufibrin matrix: AFM
regenerative endodontic treatment: RET
mobilized dental pulp stem cells: MDPSCs
extracellular matrix: ECM
periodontal ligament stem cells: PDLSCs
polylactic acid: PLA
poly(lactic-co-glycolic acid): PLGA
core layer: CL;
poly(dl-lactide-co-ε-caprolactone): PLCL;
hydroxyapatite nanoparticles: Hap;
microRNA: miRNA
self-assembling peptide hydrogel: SAPH
matrix extracellular phosphoglycoprotein: MEPE

The carious process leads to inflammation of pulp tissue. Current care options include root canal treatment or apexification. These procedures, however, result in the loss of tooth vitality, sensitivity, and healing. Pulp capping and dental pulp regeneration are continually evolving techniques to regenerate pulp tissue, avoiding necrosis and loss of vitality. Many studies have successfully employed stem/progenitor cell populations, revascularization approaches, scaffolds or material-based strategies for pulp regeneration. Here we outline advantages and disadvantages of different methods and techniques which are currently being used in the field of regenerative endodontics. We also summarize recent findings on efficacious peptide-based materials which target the dental niche.

### 1 Tooth structure

Humans have two sets of teeth: 20 primary/deciduous teeth and 32 permanent teeth [1], each composed of organized, mineralized tissue layers of dentin [2], cementum and enamel [1]. In native tooth architecture, an enamel-encased crown surrounds the live internal pulp chamber and roots [1].

**Fig. 1:**
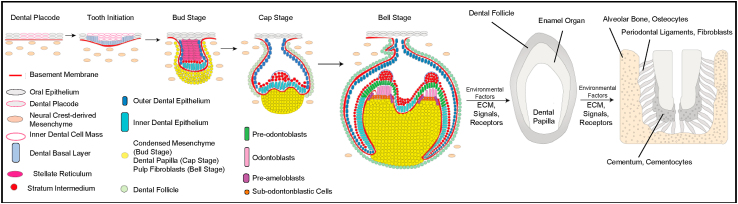
**Formation of a Tooth**. Tooth development begins *in utero* and follows 5 stages: dental placode formation, tooth initiation, the bud stage, the cap stage and finally the bell stage. Environmental factors stimulate tooth maturation further, encasing the dental papilla beneath the enamel organ and dental follicle. Finally osteocytes foster alveolar bone formation, fibroblasts generate periodontal ligaments and cementocytes deposit cementum.

Enamel is derived from oral epithelium tissue, while dentin, pulp and periodontium derive from the neural crest [1,2] (Fig. 1)

In healthy tooth anatomy, the dentin-pulp complex lies below a continuous layer of ordered enamel, protecting the vessel- and nerve-rich pulp [1]. In the dentin layer, odontoblasts create and regulate tissue matrix components [2,5]. Epithelial-mesenchymal interactions are essential for the transition of mesenchymal embryonic pulp cells to the pre-odontoblastic stage [5]. Signaling molecules from the inner enamel epithelium encourage differentiation of peripheral dental papilla cells, odontoblast precursors, which eventually become secondary odontoblasts [5].

Human dental pulp stem cells (DPSCs) originate from migrating neural crest cells, are derived from the embryonic ectoderm layer and possess mesenchymal stem cell properties [9]. This feature confers them vast differentiation potential, in addition to their ability to secrete trophic factors and their immunoregulatory properties [10]. DPSCs can differentiate into odontoblasts, osteocytes/osteoblasts, adipocytes, chondrocytes, or neural cells [10]. DPSCs can also regenerate dental tissue composed of vascular, connective, and neural tissues [10]. During tooth development, primitive ectomesenchyme becomes enclosed within the prospective teeth to form the dental pulp, a rich source of stem cells [8]. Odontogenesis (the process of tooth development) involves the matrix of cell types and specific cellular processes which result in the differentiation, growth, maturation and eruption of developing teeth in the mouth [1].

### 2 Tooth pathology

When tooth structure is endangered by pathologies such as caries, odontoblasts attempt to seal off dentinal tubules to protect the pulp [1]. The carious process gives rise to the formation of porous lesions which expose and damage organic material which lies below the enamel [2]. At the plaque-enamel border, for example, acids secreted by bacteria demineralize enamel [3] and creates pores of increasing size on the tooth surface; breached enamel leads to pulp involvement *via* tubular fluid and odontoblastic processes, and requires endodontic intervention [3]. Deep caries with pulp or near pulp involvement are often treated with medical paste or pulp capping to prevent further inflammation and prevent bacterial invasion [4].

The most common cause of traumatic dental injuries is sport-related activity-as participation in such sporting activities has increased over the last decade, so has the frequency of such injuries [5], making this type of injury a recognized public health problem worldwide. In the United States specifically, almost every third child with primary teeth and every fourth adult has evidence of traumatic dental injuries [5]. Traumatic dental injuries are often detrimental for odontoblasts in close proximity to the lesion site, and their cell death triggers activation of dental pulp stem/progenitor cells [6]. Although the exact stored location of these mesenchymal cells is not yet known, this type of damaging event causes these cells to proliferate, migrate and differentiate [6].

Other pathologies that require endodontic intervention include but are not limited to: pulpitis, pulp necrosis, and apical periodontitis including acute and chronic apical abscesses [7]. Apical periodontitis is inflammation caused by a diverse category of microbiota outside necrotic root canals (primary), or improperly treated root canals with persistent infection (secondary) [8]. Both primary apical periodontitis (PAP) and secondary apical periodontitis (SAP)-causing bacteria exacerbate other systemic diseases in patients. Although approximately 200–300 bacterial species can be cultured from samples collected in the oral cavity, only few of these species have been isolated from necrotic root canals [9]. This area is populated by strictly anaerobic bacteria. The identification of bacterial taxa differentially abundant in primary and secondary infections may provide a basis for targeted therapeutic approaches aimed at reducing the incidence of apical periodontitis [10]. If bacteria from the oral microbiome gain systemic access, this leads to systemic ailments such as bacteremia, endocarditis and atherosclerosis [11].

### 3 Treatment options

Currently root canal treatment and apexification are 2 standard treatment options available in the clinic [12]. Choice of procedure depends on the stage tooth development. Apexification is appropriate for immature permanent teeth with open apexes, whereas root canal procedure is more suitable for mature teeth with closed apexes [13]. Application of these techniques, however, results in the loss of tooth vitality [14]. Conversely, if the pulp is partially vital with no inflammation present, then irreversible pulpitis, pulpotomy and pulp capping procedures can be considered to treat exposed pulp [14,15].

#### 3.1 Conventional root canal treatment

**Fig. 2:**
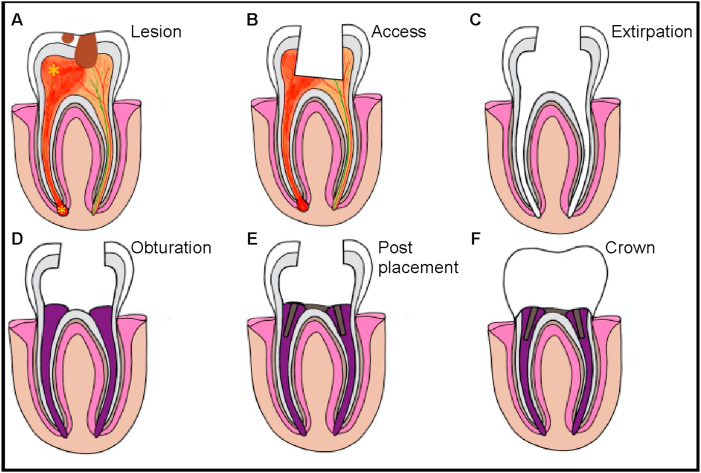
**Endodontic Treatment of an Apical Tooth Abscess with Concurrent Caries**. **A**) A diseased tooth with caries results in soft tissue inflammation (*) and damage to tooth enamel, exposing the pulp. Initially **B**) access to the pulp is obtained to **C**) extirpate inflamed and necrotic tissue and disinfect the tooth cavity. **D**) Obturation fills the emptied tooth, typically employing inert materials such as gutta-percha. Treatment is completed with **E**) post and core installation and finally **F**) placement of an artificial crown to form a protective barrier.

Conventional root canal procedures (Fig. 2) [12] remove inflamed pulp and repair root canal structure, rather than focusing on tissue regeneration [16], have been standard treatment options and have been well-described elsewhere [[17], [18], [19], [20]].

Materials and techniques used in the disinfection, filling or sealing processes affect the overall success of the procedure [21,22], and today many different disinfectants (Table 1), core materials and sealers (Table 2) are available to clinicians when performing traditional root canals [23].

**Table 1:** A comparison of current disinfectants used in endodontic procedures.

Material	Advantages	Drawbacks	References
Sodium Hypochlorite (NaOCl)	bactericidal tissue dissolution	not biocompatible inactivates dentin-matrix components altered tissue mechanical properties	12, 28, 29
Saline	95% bacterial reduction when used with instrumentation	insufficient sanitization	30
EDTA/Citric Acid	growth factor solubilization dentin formation antimicrobial	dentin erosion	28, 30
Chlorhexidine (CHX)	highly effective against yeast, gram-positive and gram-negative bacteria	lack of tissue dissolving properties	30
MTAD and Tetraclean	easy removal of smear and organic layer in infected canals	does not treat infection	30
Calcium Hydroxide	strongly antimicrobial	cytotoxic upon long exposure	30
Triple Antibiotic Paste (TAP)	strongly antimicrobial	high concentration lowers cell survival and proliferation	30,31

**Table 2:** Commonly used materials categorized by their procedure type.

Procedure	Material	Advantage	Disadvantage	References
Obturation	Gutta-Percha	stable biocompatible low toxicity	low adhesion microleakge	32–34
	Resin	good seal formation tooth strengthening	limitations in physical and mechanical properties	32
Apexification	Calcium Hydroxide (CH)	strenthens immature roots	long application process lowers mechanical strength of tooth	29
	Mineral Trioxide Aggregate (MTA)	biocompatible sterile one-step superior sealing ability stimulates high quality/quantity dentin	expensive best suited for revascularization	29, 32, 35
Pulp Capping	Calcium Hydroxide (CH)	bactericidal promotes odontoblast differentiation high pH	poor bonding to dentin can cause secondary inflammation	36–38
	Mineral Trioxide Aggregate (MTA)	bactericidal low solubility good sealing properties thick and fast dentin-bridge formation biocompatible	expesive long setting time tooth discoloration poor mechanical properties	36,37
	Biodentine	dentin-like mechanical propertiesorganized odontoblast layers stimulates dentin-bridge formation	not signficantly different from MTA	39,50

While roots canals show a high success rate in treating apical periodontitis, teeth that undergo root canals lose vitality and important functions like dentin formation, and root thickening, lengthening and maturation [12,24], giving rise to teeth which are non-vital, brittle, prone to reinfection, and susceptible to fracture [14]. A retrospective study showed treatment success is highly dependent on the correct canal filling length and the position of the treated tooth, with posterior teeth showing greater healing rates than anterior teeth [25]. When this endodontic procedure fails, other treatment options include revision, apicoectomy or finally, extraction. This is generally not preferred because of negative effects on oral health and quality of life [26,27]. Improper or incomplete extraction of the tooth also results in physical, financial, and emotional burden to the patient [28].

#### 3.2 Apexification

Apexification offers another strategy to treat immature dental pulp with open apexes (without apical closure) [15,29,30]. In this technique, a mineralized barrier, such as CH or MTA, is placed near the apex of the root for the closure [[29], [30], [31]]. MTA is preferable to materials such as CH for this step as it has few negative effects on dentin anatomy and performance, does not require multiple follow-up clinical visits and has a significantly higher success rate [15,32]. MTA plugs, however, are markedly more expensive, and overall apexification as a treatment option does not show great potential for root maturation or immunity, both required for tooth vitality and development [15,[29], [30], [31]]. Because of this, vital pulp therapies offer an attractive alternative to preserve tooth functionality while preventing the tooth loss.

#### 3.3 Vital pulp therapy and apexogenesis

Vital pulp therapy (VPT) treats teeth with partially vital pulp or reversible pulpitis [33] and maintains pulp tissue vitality and root maturation [34], making it more desirable than traditional root canal therapy. Apexogenesis is a similar technique to apexification (applied on necrotic pulp) and can only be applied to the immature permanent teeth with open apexes (without apical closure) and with some remaining vital pulp [15]. In apexogenesis, the inflamed pulp tissue is removed, and an apical barrier agent (typically CH or MTA) is applied along healthy portion of the pulp [13,15]. Final outcomes include root, and dentin-bridge formation, and continued physiological tooth development, although these results may take up to 2 years to fully realize [13].

#### 3.4 Pulpotomy and pulp capping

Direct pulpotomy and pulp capping treat exposed vital pulp while avoiding necrosis [14]. A pulpotomy removes infected, inflamed pulp, prevents the spread of infection and ensures the health and function of unaffected pulp [35]. If tooth inflammation is not severe, pulp capping is used to establish a protective barrier, protecting the tooth interior, maintaining pulp vitality, fostering healthy regeneration and dentin bridge formation [36]. Pulp capping is either direct or indirect; in indirect pulp capping, pulp tissue is not exposed, and a biomaterial is applied to the thin dentin layer already present [35]. In direct pulp capping, the biomaterial is applied directly as an interface against the remaining exposed vital pulp tissue, creating a seal to prevent further exposure to the oral environment. Similar to indirect pulp capping, direct pulp capping helps maintain pulp integrity and vitality, and facilitates tertiary dentin formation [[35], [36], [37]]. Pulp capping is well-suited for immature permanent teeth, as it helps stimulate root maturation, an outcome not observed in apexification [37]. Ultimate treatment success, however, is highly dependent on the capping material [24]. The final deciding factor between pulpotomy and pulp capping is related to infection severity, with more severely infected teeth undergoing the former [36]. Despite their potential, insufficiencies still exist in current treatments, including a lack of data on long-term efficacy [38], reliably preventing bacterial contamination, minimizing scar tissue formation [24], and the formation of new dentin structures which are imperfect or irregular [14]. Many of these shortcomings are being addressed by newly emerging biomaterial strategies, discussed later.

### 4 Dental tissue regeneration

Dental pulp regeneration is a new and developing technique for dental procedures aimed at revitalizing infected, necrotic or lost dental pulp to restore natural functions such as mineralization, pulp immunity and sensitivity [39]. This technique incorporates and balances 3 main components: cells (mostly stem cells), bioactive molecules (generally growth factors), and scaffolds.

Regenerative signals can originate from growth factors, the scaffold, plasma or cells such as dentin/odontoblasts, pulp fibroblasts or endothelial cells [40]. The release of dentin-originated bioactive molecules is stimulated by bacterial acids produced during caries-restoring procedures, or the placement of MTA or calcium hydroxide agents during pulp capping [40,41]. The secretion of bioactive molecules and growth factors by odontoblasts and their incorporation within the dentin extracellular matrix (dECM) leads to dentin production (dentinogenesis). Some growth factors utilized in dECM by calcium hydroxide, white MTA, and grey MTA include VEGF, FGF-2, PDGF, transforming growth factor-beta 1(TGFβ-1), and insulin like growth factors (IGF-1, IGF-2) [40,41]. Pulp fibroblasts and endothelial cells are other sources of growth factor release for specific tasks such as cell migration, proliferation, differentiation, and angiogenesis. In addition, the plasma signal C5's successful complement activation to regulate inflammatory reactions shows that it could serve in dentin-pulp regeneration signaling [40].

Newly regenerated dentin-pulp tissue must be similar to the original tissue, which consists of well-organized connective dentin tissue and live vascularized, innervated pulp. Currently, most studies focus on revascularization and dentin deposition of new tissue [42]. Revascularization procedures show promising results for immature teeth and are easier to apply in the clinic. Tissues formed in the root canal with this procedure, however, are not consistently representative of native true dentin-pulp complex [16,43]. Studies still struggle to regenerate a new pulp which is morphologically and functionally similar to natural pulp [29]. In addition to vascularization and proper soft tissue regeneration, success criteria for the regeneration includes observing remineralization, cell-matrix interactions, innervation, growth factor incorporation, controlled bio-degradation, and pathogen control and mitigation in the regenerated tissue [24]. Observation of tooth changes such as apical closure, root lengthening, radiographic criteria, and dentin wall thickening suggest improved root maturation, dentinogenesis (formation of dentin), and wound healing [29,31,44]. Additionally, the direct availability of the cell constructs is important, especially for older patients who may not have enough autologous cells to recruit [16,29,31,43,45,46].

As of today, there are several main approaches used to achieve dentin-pulp regeneration [42], the first is cell-based therapy, or cell transplantation. *Via* this method, many different cells (mostly stem cells) are isolated and cultured *in vitro*, and then placed in an appropriate scaffold to be inserted into the root canal [42]. Similarly, a second cell-guided route to generate dentin-pulp complex is through endogenous regeneration, or cell homing. In this method a specialized niche is created at the injury site for host cell mobilization and homing. This site is also amenable to native cell proliferation and differentiation for repair [47]. Kang et al. at the University of California are conducting a clinical trial in which mesenchymal stem cells are implanted within the dental cavity to assess its angiogenic potential in dental pulp revascularization; one of many on-going clinical trials for dental regeneration (Table 3). To avoid the complications of harvesting and maintaining one or multiple cell types, some methods employ growth factors to promote the migration, proliferation, and differentiation of local stem cells. Much recent research in the field of hard and soft tissue dental regeneration has focused on the use of materials, such as traditional and bioceramics, naturally derived biomaterials and scaffolds, and synthetically prepared materials, all matrices which themselves can be used or tuned to serve as origins of regenerative signals. Exciting new research in synthetic biomimetic materials recapitulates aspects of each of these materials, giving rise to simply formulated sophisticated materials to guide hard or soft tissue regeneration. Below we discuss each of these approaches in detail, and for each category highlight recent work regarding tissue regeneration in the dental niche.

**Table 3:** Clinical trials and their strategies to treat dental pathologies.

Strategy	In vivo	Clinical Trial	Reference/University
Tissue transplantation	50 participants (7–50 y.o.)	N/A	UCLA School of Dentistry
Apexification/revascularization	30 participants (7–25 y.o.)	Phase 4	University of Liverpool, UK
Pulp necrosis with Biodentine & MTA	26 participants (8–15 y.o.)	N/A	Cairo University, Egypt
Pulp necrosis	80 participants (7–12 y.o.)	N/A	Fourth Military Medical University, China
Pulp necrosis with MTA, double antibiotic paste & triple antibiotic paste	10 participants (7–60 y.o)	Phase 1	The University of Texas Health Science Center at San Antonio
Revascularization with antibiotic paste and MTA	30 participants (7–25 y.o.)	Phase 4	University of Liverpool, UK
Revascularization with triple antibiotic paste, ciproflocacin/propolis, ciprofloxacin/metronidazole, and propolis/metronidazole	40 participants (8–18 y.o.)	Phase 4	Ain Shams University, Egypt

#### 4.1 Stem cell-based therapies

Many cell types have been used successfully in cell-based pulp regeneration studies [39,[48], [49], [50], [51], [52], [53], [54], [55], [56], [57]] (Fig. 1). Adult mesenchymal stem cells (MSCs) are common as they can differentiate into many specialized tissues and cell types which are crucial for maintaining tooth homeostasis, including odontoblasts (cells that produce dentin), chondrocytes, myocytes, and adipocytes [12,36]. Most (though not all) stem cell populations in the tooth share properties of bone marrow-derived mesenchymal stem cells, also called dental mesenchymal stem cells [58]. Five dental stem cells involved in tooth formation are: DPSCs, stem cells from human exfoliated deciduous teeth (SHED), stem cells from the apical part of the dental papilla (SCAP), periodontal ligament stem cells (PDLSC), and stem cells from the dental follicle (DFSC) [36], all named according to their tissue of origin [58]. DPSCs, SHED and SCAP are especially crucial in pulp regeneration studies since they are derived from native pulp or precursor tissue [36]. In addition, when dental epithelial stem cells (DESCs) are combined with dental mesenchymal stem cells, the mixed population together can regenerate a dentin-enamel-like complex structure [12].

When using DPSCs for dentin pulp tissue regeneration, the effect of appropriate growth factors must also be investigated and understood. Growth factors are released from multiple sources, including stem cells themselves, dentin, other cells, or scaffold materials, all of which work together to regulate the behavior of immature undifferentiated DPSCs [11]. Growth factors induce cell proliferation, angiogenesis, and neovascularization, all essential steps in the tissue regeneration process [[11], [12], [13]]. Signaling molecules work in together with chemotactic agents and other signaling factors to attract stem cells to the defect site in need of repair and stimulate local regeneration [12,13]. These polypeptide growth factors mediate a wide range of functions, such as enhancing DPSC migration through 3D collagen gels (stromal cell-derived factor-1 SDF-1 and basic fibroblastic growth factor bFGF) and odonto/osteogenic differentiation (bone morphogenic protein BMP7) [13].

Pulp regeneration is specifically associated with vascular endothelial growth factor (VEGF), bFGF, platelet-derived growth factor (PDGF), nerve growth factor (NGF), and BMP7 [12,13]. VEGF plays a critical role in angiogenesis and revascularization as it binds to heparin and increases endothelial cell proliferation and neovessel formation [12,13]. bFGF has angiogenic potential and recruits DPSCs to migrate and proliferate without differentiating [13]. Platelets release PDGF which is important in cell proliferation and angiogenesis [12]. PDGF can significantly enhance DPSC proliferation and odontoblastic differentiation [14]. NGF expression is high during tooth development and at areas of tooth defects, when it aids in the survival and proliferation of sensory and sympathetic neuronal cells [14]. Finally, BMP7 induces dentin formation (dentinogenesis) [12].

Gronthos et al. utilized MSC-like stem cells from dental pulp tissue obtained from human third molars, termed dental pulp stem cells [59]. Distinct advantages of SHED over DPSCs include a higher proliferation rate and enhanced differentiation potential. Sonoyama et al. confirmed that SCAP arise from the soft tissue at the tooth apex [60].

Besides dental stem cell-based approaches, non-dental stem cells are also used in tooth and periodontal tissue regeneration, including bone marrow-derived mesenchymal stem cells (BMMSCs), adipose-derived stem cells (ADSCs), embryonic stem cells (ESCs), neonatal stem cells from the umbilical cord, and induced pluripotent stem cells (iPSCs). iPSCs’ ability to differentiate into mesenchymal stem cells and osteoprogenitor cells makes them an attractive choice for dental tissue regeneration [12,36]. Additionally, as they are produced by adult somatic cells (which cannot further differentiate back to a pluripotent condition), iPSCs are a good alternative for older patients who no longer have sufficient pulp tissue for regeneration [29].

**Fig. 3:**
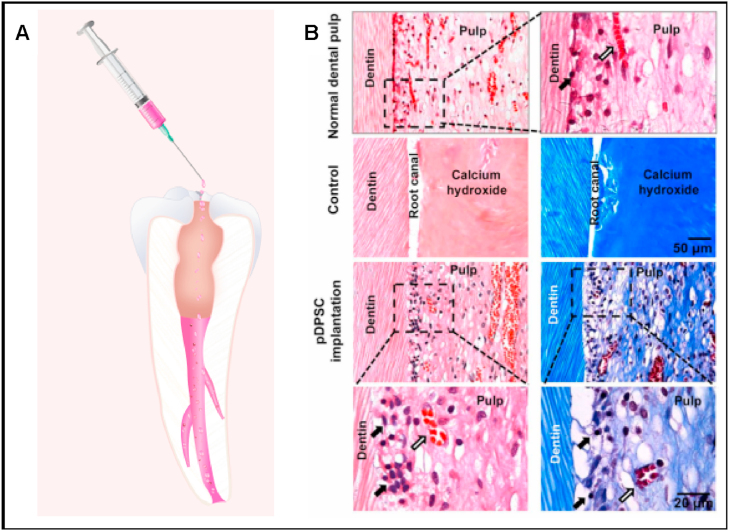
**Procedure and histological analysis of pig DPSCs implanted into minipigs**. **A**) Pig DPSCs (pDPSCs) were implanted into permanent incisors of minipigs after pulpectomy (n = 3). **B**) H&E staining (left) and Masson staining (right) demonstrate pulp tissue regeneration 3 months after pDPSC implantation. As a control, CH instead of pDPSCs was inserted into young permanent incisors in minipigs (n = 3). After 3 months, no pulp tissue was regenerated and only calcium hydroxide was observed. Normal pulp tissue of minipigs was stained for comparison (top). Scale bar, 50 μm. Enlarged images show odontoblasts (black arrow) and blood vessels (open arrow) in select regions of regenerated pulp tissue. Scale bar, μm. (Panel B is adapted from Xuan et al [66] (C) AAAS). Reprodcued with permission [66]. 2018, Sci. Trans. Med.

For *in vivo* observation of pulp-dentin regeneration, many different animal models are used to test cell-based therapies [61]. Initial studies were conducted on small animals such as mice and rats due to their accessibility [46]. Larger animal studies, however, such as those that employ dog and swine models, provide an environment more similar to human oral tissue [46]. Scaffolds based on various natural, synthetic or hybrid materials have been used carriers in these studies [62]. The scaffold creates a supportive environment for stem cell delivery to the pulp, and can provide or mimic growth factors to enhance and guide differentiation [62,63]. Cordeiro et al. observed the successful differentiation of SHEDS into odontoblast-like and endothelial-like cells *in vivo* by transplanting the cells into immunocompromised mice within a biodegradable scaffold [64]. Zhang et al. used a composite of hydroxyapatite (HA), silk fibroin (SF) and ultra-small super paramagnetic iron oxide (USPIO) as a scaffold for the delivery of DPSCs [65]. HA and SF were biocompatible, biodegradable, had desirable mechanical properties and fostered DPSCs proliferation and osteoinduction *in vivo* to regenerate dental pulp tissue [65]. Additionally, because of their paramagnetic properties, USPIO could be used for noninvasive imaging [65]. Gronthos et al. demonstrated successful dentin/pulp-like tissue regeneration *ex vivo* using DPSCs embedded in a hydroxyapatite/tricalcium phosphate (HA/TCP) scaffold; transplantation of their material into 10 wk old immunocompromised mice gave comparable results to controls employing bone marrow stromal cells (BMSCs) [59]. Xuan et al. inserted DPSC aggregates into the root canals of human teeth and implanted the root subcutaneously into female immunocompromised mice for 8 weeks; notably this study observed the *in vivo* differentiation of DPSCs into sensory neurons [66]. In another study, a copolymer of a poly-D,l-lactide and glycolide (PLG) scaffold included SCAPs and DPSCs, and was subcutaneously implanted to female severe combined immunodeficient mice (6–8 wk old) for 3–4 months. After explanting, a continuous layer of dentin-like tissue was observed on the canal dentinal wall. At the study conclusion, well-vascularized pulp-like tissue regenerated in the root canal space [67]. Large animal models such as swine have also been used to develop pulp and dentin regeneration strategies. In one study, dentin regeneration was achieved through mixing porcine deciduous pulp stem/progenitor cells (pDPSCs) with a β-tricalcium phosphate (b-TCP) scaffold [68]. Xuan et al. isolated and implanted DPSCs into the empty root canals of female minipigs *in vivo* and saw vascular, innervated tissue regeneration within the odontoblast layer (Fig. 3) [66].

Similarly, Xuan et al. conducted a study on young patients (aged 7–12) with injured teeth using a cell-based method and electrical pulp tests to assess the pulp vitality [66]. The periapical tissue was probed to induce bleeding and induce subsequent clot formation in the apical foramen. Successful regeneration of ordered 3D pulp tissue with new blood vessels and sensory nerves was observed, and promisingly, in follow-up studies, closure of apical foramen and elongation of roots were noted [66].

In a different study, autologous pulp stem/progenitor (CD105+) cells and stromal derived factor-1 (SDF-1) were combined in a collagen scaffold and transplanted into canine root canals [91]. After 14 days, the CD105+ cells expressed angiogenic and neurogenic factors, and regeneration of pulp tissue was seen [69]. Similarly, Itoh et al. prepared DPSC constructs by shaping sheet-like aggregates of DPSCs with a thermoresponsive hydrogel and showed stem cells within the constructs remained viable after prolonged culture [48]. Pulp-like tissues rich with blood vessels formed within the human tooth 6 week post subcutaneous implantation in immunodeficient mice [48]. The authors also noted DPSCs in contact with dentin differentiated into odontoblast-like cells [48].

For patients who do not possess enough native tissue for endogenous MSCs, recent methodologies have developed induced MSCs (iMSCs) which led to the acquisition of stem characteristics and an epithelial-mesenchymal transition [39]. These cells generate normal human epidermal keratinocytes (NHEKs) through an epithelial-mesenchymal transition [29]. Although all of these studies give promising results for the regeneration of pulpless teeth, this procedure is challenging to apply under clinical conditions [48,70]. Some current challenges still facing cell-based therapies include the high expense of current good manufacturing practice (cGMP) facilities, a lack of information in the scientific community regarding the outcome of allogenic dental MSC for pulp/dentin regeneration, a lack of a centralized dental stem cell banking system, and a lack of recognition and practice of cell-based pulp/dentin regeneration therapies by the medical field [71]. In addition, these complicated procedures remain more difficult to obtain procedural approval [70].

Stem cell sheets have been explored as an alternate strategy to promote dental pulp regeneration. Hu et al. cultured and fabricated three stem cell sheets from cell types located within and around the vital pulp: DPSCs, periodontal ligament stem cells (PDLSCs) and SCAPs [72]. Several *in vitro* assays were performed to determine biocompatibility and the stemness of these sheets including RT-PCR to evaluate OCT4, SOX2 and TERT expression. Further, the authors stained for various marker genes such as collagen type-1 and fibronectin. All 3 sheets retained the expression of these markers and there was no distinguishable difference between the scaffolds in signaling. The 3 stem cell sheets were then implanted subcutaneously in 10-week-old immunocompromised binge mice for 8 weeks, explanted and processed for histology. The SCAP stem cell sheet displayed significantly greater mineralization and fibronectin expression compared to the DPSC and PDLSC sheets [72]. This strategy, while time consuming, provides encouraging alternative solutions for mineral tissue regeneration.

In a cell-homing technique, instead of an exogenous scaffold, a blood clot is created within the pulp canal to itself act a scaffold [71,73,74] and recruit endogenous cells *via* native growth factors (GFs) [42,71]. Fibroblasts and fibrocytes are the greatest contributors to the regenerative response and GF expression. Duncan et al. observed that the revitalization of pulp-like tissue is possible with the release of selected exogenous GFs with transplanted stem cell scaffolds [75]. Kim et al. used bFGF, VEGF, PDGF, NGF and BMP-7 to promote angiogenesis and mineralization after 3 weeks. In their study, teeth which had already undergone endodontic treatment were implanted subcutaneously filled with either a cytokine-loaded or cytokine-free collagen gel into 5–7 week old male mice [73].

Blood clot formation for revascularization is the most common of the cell-homing strategies applied clinically for dentin-pulp regeneration [32,66]. In 2011, the American Dental Association (ADA) approved the use of apical revascularization (AR) as a new treatment modality [99]. The regeneration of pulp is stimulated by localizing blood into the entire root canal. This procedure is applied either by over instrumentation or using platelet-rich plasma (PRP), platelet rich fibrin (PRF) or autologous fibrin matrix (AFM) [76]. Over-instrumentation is a strategy in which a blood clot is induced to form a fibrin-based scaffold, and has the highest effectiveness in the adult population [54]. Although this technique is generally used to treat pulp necrosis, it can only be used for immature teeth with open apices and is only acceptable for teeth with completely developed roots [99].

**Fig. 4:**
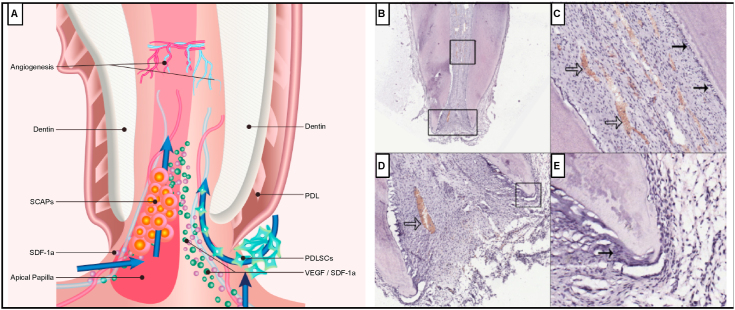
**Schematic representation and histologic observation of a human immature permanent tooth with irreversible pulpitis after revascularization/regeneration procedure**. **A**) Migration of cells (PDLSCs, SCAPs) and growth factors (VEGF, SDF-1a) into the tooth interior promotes angiogenesis in the tooth cavity. **B**) Histology of an extracted revascularized tooth, from which the MTA plug was removed prior to histological tissue processing. Connective tissue and collagen fibers fill the canal space. **C**) A higher magnification image of the square in B showing the apical root canal. Solid arrows indicate flattened odontoblast-like cells lining the predentin, and open arrows reveal the presence of many blood vessels filled with red blood cells. **D**) A higher magnification image of the rectangle in B showing the apical foramen. The presence of blood vessels is indicated by arrows. **E**) A higher magnification image of the square in D showing part of the root apex. Arrows indicate layers of epithelial-like HERS surrounding the root apex. Reprodcued with permission [78]. 2012, J. Endo.

Periapical radiography with paralleling can help monitor root development [77]. Although some clinical studies showed positive results using sensibility test after regenerative endodontic treatment (RET), there is limited histological tooth data [36,77]. Without any histological analysis, regeneration cannot be observed or confirmed radiographically. Shimizu et al. conducted the first study to determine the histological results of regeneration/revascularization in the root canal of necrotic immature permanent human teeth with irreversible pulpitis (Fig. 4A) [78]. The approach proved successful and at 3.5 weeks after the revascularization procedure, loose connective tissue and collagen fibers were observed in the canal (Fig. 4 Panel A). Spindle-shaped fibroblasts or mesenchymal stem cells were observed at the periapical area, as well as blood vessels and cellular components inside the canal (Fig. 4 Panel B). Also, odontoblast-like cells were observed along the pre-dentin and root apex surrounded by epithelial-like cells. No nerve fibers were observed however (Fig. 4 Panel B) [78].

**Fig. 5:**
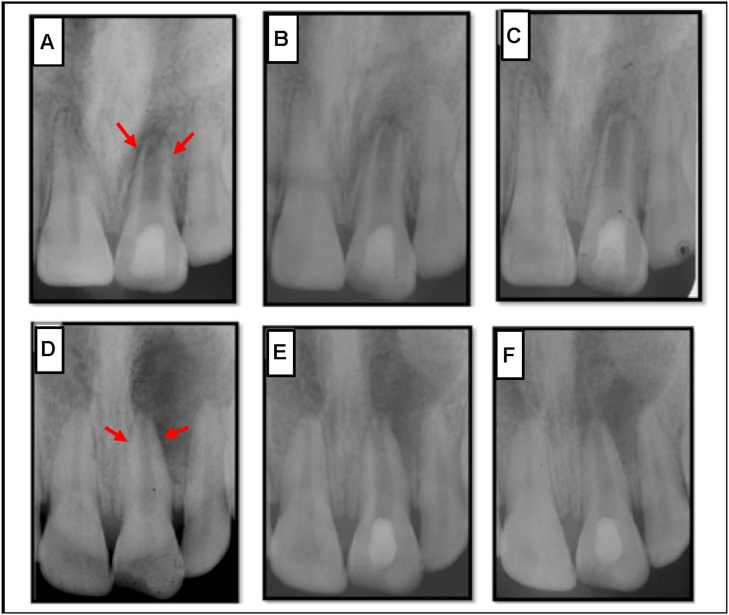
**X-ray of teeth with revascularization**. **A-C**) Show teeth with revascularization. **A**) The dentinal walls (red arrows) for this patient, a 9 year old girl, are thin with a larger opening at the apex. **B**) after 6 months, there is calcification present at the apex. **C**) After 1 year, revascularization of the tooth is achieved, primarily through the bridge composed of calcium at the apical section and root lengthening. **D-F**) Show teeth with revascularization and PRP. **D**) Similar to above, the dentinal walls (red arrows) are thin with a larger opening at the apex for this patient, a 15 year old boy. This patient underwent a treatment an identical procedure however supplemented with PRP. **E**) At 6 months, the calcium barrier was reduced at the apex compared to the 9 year old girl and the patient reported to be symptom free. **F**) After 1 year, revascularization of the tooth is successful and is comparable to a normal apical tooth. Reprodcued with permission [44]. 2021, J. Endo.

By bleeding induction, MSCs can be delivered into the root canal space and irrigators such as EDTA can promote growth factor release from dentin [29]. In a pilot clinical study, mobilized dental pulp stem cells (MDPSCs) were transplanted into 5 patients; 3 showed successful dentin formation, though further extended studies are still required to allow dentin to fully cover pulp tissue and prevent microleakage [79]. Clinical study shows that when revascularization is supplemented with a PRP scaffold carried on a collagen sponge, a better healing process is observed than in the induced revascularization group (Fig. 5) [44]. This might be due in part to specific advantages that the PRP provides, including growth factors, anti-inflammatory agents, cell differentiation signals and the ability to modulate the inflammatory response [35].

While revascularization is easier to perform in a clinical environment compared to a stem-cell based therapy, it still has some inherent limitations [70,71]. The successful release of growth factors depends on many different elements, including disinfection or rinsing after endodontic access and the total migration of stem cells [75,[80], [81], [82], [83]]. In addition to growth factor release, to protect the thin root canal walls and stem cell vitality at the apical tissue for eventual root maturation, lower concentrations of disinfectants (mostly NaOCl and EDTA) and intracanal medicaments (TAP or CH) are preferred for regenerative applications [29]. Some studies show, however, that this causes incomplete disinfection [29]. Having too few cells recruited may also affect or impede the root development, and cause insufficient bleeding [29]. Besides these complications, to date, the observation of revascularization is associated with regeneration of the entire dentin-pulp complex. Despite these promising lead results, further studies need to be conducted to develop robust methods to properly deliver signaling molecules and regenerate an organized 3D pulp structure [36].

#### 4.2 Cell and materials based strategies

##### 4.2.1 Endodontic treatment using traditional ceramics

While cell- and growth factor-based strategies show good promise for regenerative endodontics, materials-based strategies have traditionally pervaded clinical applications, with the majority of these to date based on ceramics and geared toward apexification, apexogenesis and pulp capping [84]. Generally these materials have a high pH to help neutralize the low pH environment of the oral cavity [35]. CH and tricalcium-silicate materials, especially MTA, are the 2 most often used in the clinic [85]. CH is desirable because of its reliability, bactericidal properties and ability to promote odontoblast differentiation. It has known drawbacks, however, including poor bonding, long-term failure, tunnel defects, and incomplete sealing resulting in microleakage [35]. Pulp capping studies with MTA have also been successful [37] because of MTA's high biocompatibility and antibacterial properties. This material sets in the tooth with a significant hardness and then presents with a low solubility [35], giving it better long-term outcomes in clinical studies compared to CH [85]. Additionally, Tomson et al. studied the effects of pulp capping agents on bioactive molecule release and observed MTA releases more bioactive molecules than CH, helping partially explain better patient outcomes with MTA treatment [41]. In pulp capping, MTA is also associated with the formation of a thicker layer of odontoblasts in the dentin bridge [35]. Known disadvantages of MTA include its high cost, tendency for discoloration of the tooth, and weak mechanical properties, features which are being addressed and improved upon by current materials research [35,85]. A recent alternative to MTA in pulp capping, Biodentine is a bioactive tricalcium silicate [86] with dentin-like properties. When in direct contact with vital pulp tissue, it facilitates the generation of dentine. A comparative study between Biodentine and CH showed Biodentine was more effective in creating an extended, thick and homogenous dentine-bridge, resulting in a better barrier to completely seal tissue pulp [87]. Biodentine shows a promisingly high success rate (82.6%) as a pulp capping agent, though patient age does affect the observed outcome [86].

While there have been significant advances improving the cytocompatibility of glass and ceramic biomaterials for regenerative endodontics, there is still a need for pulp capping agents which can facilitate new tissue growth. Tailored amorphous multiporous TAMP scaffolds, composites of calcium oxide and silicates, are a promising new class of material which has demonstrated robust regeneration and preservation of bone and soft tissues [[88], [89], [90], [91]], and more recently shown good biocompatibility as a pulp capping agent with human and swine DPSCs *in vitro* and with pulp *in vivo* [92]. After 4.5 month testing in mini-swine, the presence of remaining TAMP and new mineralized dentin bridge tissue has formed in all cases, and is the first instance of this type of material being tested for dental applications to be followed up by further large animal *in vivo* studies [92].

##### 4.2.2 Naturally derived biomaterials and scaffolds

A notable disadvantage of both the new and traditional approaches outlined above is the lack of extracellular matrix (ECM) or ECM-mimetic scaffolds to support cell proliferation and differentiation, crucial to long-term tooth vitality and tissue homeostasis. Materials-based strategies that combine the previous approaches, or which confer inherent bioactivity to guide regeneration, will prove most efficacious long-term, and below we highlight timely examples which harness naturally derived scaffold materials.

In these applications, naturally derived materials are often synthetically modified or prepared as material composites to improve and tune their physical and biological properties. In one recent example, xenograft equine bone hydroxyapatite modified with a poly(E-caprolactone) was generated to recapitulate the morphological and biochemical features of native bone, and notably did not induce infection or immune response [93]. The authors report that an increase in the bioceramic content improved calcium deposition, cell viability and osteogenesis [93]. Electrospinning of these solutions generated aligned mats of nanofibers which were better able to promote osteogenic differentiation in DPSCs than controls [93], in line with previous reports that show aligned structures significantly improve cell adhesion and proliferation [[94], [95], [96], [97]]. In a similar report, naturally derived equine bone was coated with polyethylenimine (PEI) [98]. The composite bone-PEI particles were well distributed with sizes below 500 nm, displayed a higher charge density and calcium ion concentration, and had better cytocompatibility than naked PEI. Excitingly, the bone-PEI nanoparticles could be used for successful BMP-2 plasmid delivery to promote osteogenic differentiation in DPSCs, almost twice as effectively as free PEI [98]. While many reports detail the success of synthetically prepared hydroxyapatite materials for non-viral gene delivery [[99], [100], [101], [102], [103]], this *in vitro* study is the first to employ naturally derived hydroxyapatite and paves the way for new and innovative *in vivo* designed oligonucleotide delivery agents for regenerative endodontics. As proteomics analysis has expanded, more information has become available corroborating commonalities between human and animal dentin matrix molecules, which in both stimulate cell migration, proliferation, differentiation and mineralization [104], validating these low-cost materials which are readily available.

While the rise of standardized, general treatments using materials from animal-derived sources, such as the above, will greatly improve clinical outcomes and advance the bioengineering field, many disparate fields of biomedicine and tissue regeneration are now moving instead towards patient-specific personalized treatments, an approach known to significantly improve individual patient diagnosis, treatment and outcome [[105], [106], [107], [108], [109], [110], [111], [112], [113], [114], [115], [116], [117], [118], [119]]. Though personalized medicine is still a burgeoning aspect of regenerative endodontics [120], some groups have recently reported encouraging examples of personalized bone engineering [121,122].

In one specific example, human demineralized dentin matrix-based materials were used as bio-ink for the fabrication of patient-specific dental tissue [122]. A composite ink of demineralized dentin and a fibrinogen-gelatin mixture was developed, and the authors demonstrated an increase in relative amount of demineralized dentin improved the mechanical and handling properties of the new material, eventually generating a 3D printable construct with a minimum line with of 252 μm^122^. Excellent cytocompatibility (>95% cell viability) and robust osteogenic differentiation of DPSCs was reported. This ink, when co-printed with DPSCs and polycaprolactone, enabled the generation of 3D tooth-shaped cellular construct (2 cm height); after a 15 d culture in medium, robust mineralization was observed as a result of odontogenic differentiation inside the construct [122]. The development of materials such as this which have excellent performance in tissue regeneration and good printability (highly resolved line widths, good stacking behavior, shear thinning), vastly increase their practicality and therapeutic range.

More traditional materials such as collagen and decellularized ECM are common for pulp regeneration therapy. In particular, collagen scaffolds supplemented with dental pulp stem cells are widely studied for dental pulp regeneration. Coyac et al. developed a dense collagen hydrogel containing suspended SHED cells through plastic compression, and investigated their biocompatibility, viability, SHED metabolic activity and mineralization over 24 days *in vitro* [123]. Live/Dead staining confirmed viability and proliferation over a 16 d period. Mineralization proteins such as the alkaline phosphatase protein and the osteopontin protein were evaluated *via* Western blot, and the group noted increased expression from day 0 up to day 24 for these markers. SEM imaging after 16 days of *in vitro* culture of the collagen gels with SHED cells revealed mineralized tissue formation throughout the core of the scaffold, indicating the potential of this hybrid approach in bone formation [123].

Decellularized ECM has also been explored as a natural scaffold to regenerate vital pulp. Alqahtani et al. developed a protocol to decellularize porcine dental pulp while still maintaining integral ECM proteins including collagen type 1, dental matrix protein 1 and dentin sialoprotein (DSP) [124]. The group implanted the decellularized ECM construct in beagles with collagen sponge controls and determined the collagen sponge resulted in disorganized tissue formation. The ECM scaffold, however, exhibited strong immunostaining for DSP throughout the bulk of the implant as well as increased cell proliferation [124].

Biopolymers such as chitosan and hyaluronic acid, which are naturally derived, low-cost, commercially available and readily modified or prepared as composites, similar to the above examples, are at the forefront of many current research efforts. Recently, these types of biopolymers have shown increased sophistication in addition their established success as scaffolds for endodontic regeneration.

In one report, Ducret et al. developed a chitosan hydrogel ± fibrin, and performed *in vitro* testing to evaluate human dental pulp regeneration. The authors prepared the composite fibrin-chitosan hydrogel and altered the relative amount chitosan (0.2–1.0% w/w) as well as chemically modifying the material through acetylation (40% acetylation determined as optimal) to find preferable mechanical handling properties to support cell proliferation and differentiation [125]. Hydrogel prepared at 10 mg/mL showed optimal mechanical properties and was chosen to be seeded with DP-MSCs (cultured over a 7 days). The nanofibrous ultrastructure was evaluated in addition to Live/Dead and collagen production assays. The authors demonstrated the fibrin-chitosan composite hydrogel showed a significant improvement in antimicrobial efficacy against *Enterococcus faecalis,* supported ECM deposition, dental pulp tissue neoformation and encouraged native fibroblast-like morphology of dental pulp-mesenchymal stem/stromal cells [125].

More recently reported by Osmond and Krebs, composites of carboxymethyl-chitosan hydrogels embedded with calcium phosphate nanoparticles were prepared and tested as pulp capping agents [126]. Their material supported DPSC proliferation for up to 3 weeks, had a high storage modulus (>1 MPa), and encouraged odontogenesis [126]. To model the release of growth factors, drugs or proteins, BSA levels were monitored and showed sustained release for 1 month, suggesting their future use as depots for long-term delivery [126].

In a similar report, a composite scaffold of chitosan and gelatin (crosslinked with either 0.1% or 1.0% glutaraldehyde) was prepared and evaluated for its potential to support DPSCs, which had or had not been pre-exposed to recombinant human BMP-2 [127]. Both constructs supported cell viability and proliferation through the final 14 d time point and each revealed significant amounts of native-like biomineralization [127]. The scaffold with a lower percentage of glutaraldehyde was more efficacious at odontogenesis (evidenced through more significant expression of Osterix, IBSP and DSPP), and *in vivo* the authors report a time-dependent mineralization which was more pronounced in recombinant human BMP-2 pre-treated cell populations [127]; overall reports such as this are encouraging, and offering viable sophisticated treatment options or orofacial bone tissue engineering. Aside from these specific examples, chitosan, modified chitosan and chitosan-containing composites have well-established success rates, and many of these materials are being translated towards the clinic [128].

A popular material in many fields of biomedicine, hyaluronic acid-based materials and composites are well-understood and have become increasingly relevant in regenerative endodontics [[129], [130], [131]]. Many of these have advanced to the clinical trial stage, where they are reported to restore diminished interdental papilla and reduce inflammation in patients with peri-implantitis [132,133]. Commercially available hyaluronic acid-based hydrogels such as Restylane offer practical advantages over other established materials such as Matrigel because of increased SCAP cell viability and proliferation, and enhanced differentiation and mineralization (evaluated through ALP, dentin matrix acidic phosphoprotein-1, dentin sialophosphoprotein and matrix extracellular phosphoglycoprotein) markers by qRT-PCR [134].

It has been well-recorded for some time that molecular weight and size of assembled hyaluronic acid-based gels impacts its biological response, although most applications employ higher molecular weight species [[129], [130], [131],[134], [135], [136], [137], [138], [139], [140], [141]]. In the dental niche, low (the result of enzymatic cleavage) and high molecular weight hyaluronic acid can differentially affect adjacent cells and tissue [142]. DPSCs treated with either low, medium or high (800, 1600 or 15,000 Da) molecular weight hyaluronic acid show significant differences in proliferation, cell morphology and size, and surface marker expression [142]. DPSCs treated with low molecular weight hyaluronic acid maintain many of their characteristic phenotypic markers (CD29, MSC and DPSC marker; CD44, T cell receptor signalling; CD73, MSC and DPSC stromal associated marker; CD90, MSC and DPSC marker), as well as additional markers not observed in the control groups (CD29, MSC and DPSC marker; CD34, transmembrane phosphoglycoprotein; CD90, MSC and DPSC marker; CD106, endothelial cell adhesion molecule; CD117-, transmembrane receptor tyrosine kinase involved in the Akt pathway and cell proliferation; CD146, melanoma cell adhesion molecule; CD166, stromal associated adhesion molecule) [142]. While the majority of reports focus only on high molecular weight hyaluronic acid-based materials, the results of this study suggest the importance in understanding the effect and timing of biomaterial degradation kinetics [142]. Further evidence supporting this idea comes from reports evaluating the impact of low and high (18 and 270 kDa) molecular weight 2-aminoethyl methacrylate-modified hyaluronic acid hydrogels *in vitro* with DPSCs [136]. The degradation, mechanical properties and swelling behavior was readily tuned by molecular weight, and these gels were readily prepared by UV crosslinking, showed no cytotoxicity and helped maintain proper DPSC cell morphology and stemness (evidenced through increased expression of NANOG and SOX2 markers) [136].

Similar to the use of other scaffolds to sequester and modulate the release payloads, hyaluronic acid-based hydrogels/matrices offer excellent potential for controlled and tunable release of charged species [[143], [144], [145]]. In a recent publication from the Gomes group, injectable hyaluronic acid gels were fabricated in situ and evaluated for their potential to encourage rapid vascularization of soft endodontic tissues [137]. Incorporation of cellulose nanocrystals improved the hydrolytic and enzymatic stability of the material, and platelet lysate to support cell proliferation and viability [137]. Hydrogels were prepared through the use of a double barrel syringe fitted with a static mixer, with barrel A containing a mixture of aldehyde-modified hyaluronic acid and aldehyde-modified cellulose nanocrystals while barrel B contained a mixture of platelet lysate and hydrazide-modified hyaluronic acid; simultaneous co-injection of both materials into molds generated stable cross-linked hydrogels which could then be tested for their physical properties and their *in vitro* and *ex vivo* performance for soft tissue regeneration [137]. This fabrication method readily facilitated incorporation of additional growth factors, PDGF and VEGF, to encourage local re-vascularization; furthermore these growth factors showed improved and sustained release profiles relative to the amount of included cellulose nanocrystals, hypothesized by the authors to arise partially from the high density of charged sulfate groups which might aid in adsorption and immobilization of growth factors [137]. An *ex vivo* chick chorioallantoic membrane (CAM) assay was used to evaluate performance of these composite materials, which generally showed promising angiogenesis, and no inflammatory response [137]. In addition, the authors noted that the addition of platelet lysate increased the elasticity of the material, showed a strong chemotactic effect, and could potentially be used to control the formation of new convergent blood vessels [137]. Finally, platelet lysate doped materials showed improved stability compared to other gels; this and cellulose nanocrystals both improved the swelling properties of the resultant gels, likely improving the local substance exchange [137].

Gels are popular treatment options as their composition can be readily altered to include active pharmaceutical agents such as antiseptics, disinfectants or bioactive substances, to improve patient outcomes [113,[146], [147], [148], [149]], [150]. In an exciting recent report, a hyaluronic acid hydrogel was modified through straightforward click chemistry to promote encapsulation of a bone morphogenetic protein-2 mimetic peptide to guide osteogenic differentiation *in vitro* and *in vivo* [151]. Crosslinking and inclusion of the BMP-2 mimetic peptide did not disrupt hydrogel formation or injectability, and the modified material served as an excellent scaffold for hDPSCs [151]. Prepared through simple mixing of a tetrazine-modified hyaluronic acid and cyclooctene-modified hyaluronic acid, this crosslinked scaffold evaded enzymatic degradation and persisted longer both *in vitro* and *in vivo*, allowing for sustained localized osteogenic differentiation for over one month [151].

#### 4.3 Growth factor guided regeneration

Growth factor guided treatments have gained much recent attention and can similarly regenerate both soft and hard tissues which recapitulate native morphology, especially when combined with cell-based therapies. Growth factors can be used to stimulate or recover cell populations, as evidenced by a recent publication by Luo et al. [152]. The authors used extracted human CD146+ DPSCs which had been cryo-preserved for 3 months, and then recovered and treated the cells with basic fibroblast growth factor bFGF in order to improve their long-term performance post-thawing [152]. Treatment with 20 ng/mL bFGF significantly improved proliferation, activated the ERK pathway, up-regulated transient receptor potential canonical 1 (TRPC1) and decreased apoptosis, all while maintaining robust stemness and pluripotency of the affected DPSCs compared to controls [152]. Long-term maintenance and viability are crucial for encouraging DPSCs and related stem cells, as a delicate balance of cytokine type and timing of application can play large role in local cell behavior and final observable outcome, demonstrated by Jaukovic et al. using IL-17 and bFGF [153]. With 7 d treatment, both growth factors could be used to modulate the behavior of SHEDs and DPSCs cell populations [153]. Treatment with either growth factor was seen to affect the relative stemness of both DPSCs and SHEDs, as demonstrated by key pluripotency markers such as OCT4, NANOG and SOX2 at both the gene and protein level [153]. The combination of IL-17 and bFGF together increased CD73 expression and decreased CD90 expression, while each factor separately induced expression of IL-6 [153]. Both SHED and DPSCs show improved proliferation and clonogenicity after bFGF treatment, similar to previous results [154,155], while IL-17 treatment stimulated SHED proliferation and clonogenicity only [153]. Their results offer new evidence suggesting bFGF and IL-17 mediate stem cell properties during different stages of growth, which could be harnessed in future therapeutic systems in which treatment timing differentially impacts patient outcome.

Recent data suggests that recently discovered concentrated growth factor can be used to stimulate proliferation and mineralization of dental pulp cells [83], in addition to its known ability modulate stemness and function in bone marrow stromal cells [156], periodontal ligament cells [157], DPSCs [158] and mesenchymal stem cells [156]. Concentrated growth factor, containing many important individual growth factors including PDGF, FDF, TGF-beta, VEGF and IGF, is known to impact many cell processes important in regenerative endodontics including adhesion, proliferation, migration, differentiation and local remodeling and angiogenesis [[156], [157], [158], [159], [160]]. In their study, Tian et al. demonstrated concentrated growth factor could be used to improve the migration, proliferation and mineralization of dental pulp cells [83]. Odontogenic differentiation was evaluated via qPCR and Western blot, revealing concentrated growth factor mechanistically upregulates gene expression of DSPP, DMP-1, BSP and ALP while simultaneously increasing protein expression of ALP, BMP2, SMAD5, Runx2 and p-SMAD [83]. The effect of concentrated growth factor on direct pulp capping was tested by the authors *in vivo* in canines, and after 3 months experimental groups showed good re-calcification, pre-dentin formation and healthy odontoblasts with regular morphology in the dental pulp [83].

#### 4.4 Synthetic materials

An ideal scaffolding material for pulp regeneration supports attachment, proliferation, and differentiation of seeded stem cells, leading to eventual vascularization and innervation of pulp tissue [[161], [162], [163], [164], [165]]. Synthetic materials and naturally derived synthetic scaffolds offer high control over material properties such as degradation rate, stiffness, reproducibility, structural tunability, epitope presentation and charge density, and have been widely applied in tissue engineering applications [163].

Synthetic polymers such as polylactic acid (PLA), poly lactic-co-glycolic acid (PLGA) and self-assembling peptides can be engineered to biodegrade as new tissue forms, leaving no permanent foreign body. Functional groups in synthetic polymers can be incorporated to attract cells or bind small molecules like growth factors [166]. Sakai et al. demonstrated formation of vascularized soft connective, pulp-like tissue and new tubular dentin when SHED cells were seeded onto PLA scaffolds [166]. Additionally, Huang et al. showed the formation of pulp-like tissue formation and dentin deposition along the root canal wall using SCAP and DPSC seeded onto PLGA [166].

Biodegradable PLA supports undifferentiated dental pulp cell adhesion and shows ideal chemical composition for mature dental pulp proliferation, performing better than collagen or calcium phosphate scaffolds [166]. Numerous studies using dental pulp stem cells report poor pulp-like structure formation with irregular shapes and orientations [167]. Mooney et al., however, combined a soft tissue core with surrounding hard tissue and seeded DPSCs into a PGA scaffold, which supported native pulp-like tissue formation better than collagen gels and alginate [59].

Another materials-based approach is the tooth slice/scaffold model in which a commercially available synthetic hydrogel composed of a 16 amino acid sequence, Puramatrix, is cultured with SHED cells [168]. Promising data has shown regeneration of pulp-like tissue and new dentin formation [168]. Multilayered and 3D printed scaffolds have shown efficacy in regenerating dental pulp [168]. Bottino et al. constructed a multilayered scaffold with 1 core layer (CL) and 2 surface layers located atop and underneath the CL [168]. This poly(dl-lactide-co-ε-caprolactone) (PLCL) scaffold was electrospun with the addition of hydroxyapatite nanoparticles (HAp) to help augment bone formation. Bottino et al. has shown periodontal regeneration *in vivo* with this hybrid scaffold design [168].

Orti et al. transplanted a 3D printed hydroxyapatite scaffolds containing peptide hydrogel combined with DPSCs in an immunocompromised mice model [63]. With these scaffolds, the authors showed blood vessel infiltration, pulp-like tissue formation and DPSC differentiation [36]. DPSCs have great potential in cell replacement strategies for dental tissue engineering due to their origin, and have been effectively used in numerous *in vivo* models, specifically for dental pulp regeneration [36]. Another strategy that has seen promise is 3D printing, as demonstrated by Orti et al., where it was used to successfully minimize scaffold variability. With their material, the authors noted consistent vascularized pulp formation and osteodentin generation *in vivo*. Further research is still required, however, to fully optimize the potential of hDPSCs, and in particular to assess and improve up the varying degrees of vascularization, innervation and hard tissue formation.

In addition to the materials discussed above, carbon-based graphene oxide materials have received much attention in biomedicine for tissue engineering and drug delivery [169,170]. The Zhang and Gu labs prepared a graphene oxide-copper nanocomposite with good water solubility and tested its ability to encourage dentin-pulp complex regeneration; promoted DPSCs adhesion, proliferation, odontoblast differentiation and secretion of VEGF and glia-derived neurotrophic factor (GDNF) [171]. When HUVECs were treated with their graphene oxide-copper nanocomposite, the authors noted robust migration, tube formation and good VEGF expression again [171]. Subcutaneous transplantation into nude mice for 8 weeks showed promising growth of new dentin-pulp complex-like features characterized by vasculature and collagen deposition surrounded by mineralized dentin-like tissue [171]. Immunofluorescence of the explanted tissue confirmed both DPSC odontogenic differentiation (visualized with dentin sialophosphoprotein), angiogenesis (CD31 and VEGF signaling via Akt-eNOS-VEGF and Erk1/2-HIF-1alpha-VEGF) and neurogenesis (GAP43), showing excellent promise for this and related materials in regenerative endodontics [171].

#### 4.5 Synthetic biomimetic materials

Recent revascularization treatments like those outlined previously are used to promote angiogenesis and revolve around growth factor- and stem cell-based therapies. Currently, growth factors such as FGF and VEGF can be delivered *in vivo* to stimulate angiogenesis [172]. VEGF isoforms VEGF-A121 and VEGF-A165 are presently being used in clinical trials [172]. RNA-based techniques utilizing microRNA (miRNA) have developed efficacious drugs such as antagomir-92a, whose angiogenic effects significantly decreased toe necrosis in mice [172,173]. Sophisticated mimicry of natural angiogenic scaffolds may prove to be the most successful, particularly with the use of self-assembling peptide hydrogels with high density epitopes mimicking VEGF [172].

Moon et al. developed an antibacterial and biomimetic nanomatrix gel which releases nitric oxide to improve upon current clinical regenerative endodontic procedures [174]. *In vitro* experiments verified antibacterial efficacy, including culture-examinations of multispecies endodontic bacteria challenged with the loaded gel (to sequester antibiotics like ciprofloxacin and metronidazole in addition to nitric oxide). Based on promising results against the bacteria, the constructs were implanted into beagles and the group was able to show their self-assembling peptide amphiphiles promoted tooth revascularization and root canal maturation. The study demonstrated nitric oxide showed dose-dependent antimicrobial efficacy, which could be used in the future to improve outcomes in current regenerative endodontic procedures and clinical trials [118].

Muller et al. developed a synthetic clay-based hypoxia-mimetic hydrogel (0.15–5 wt%) co-cultured with dental pulp derived stem cells to regenerate pulp, and determined that these constructs were both biocompatible and stimulated VEGF production within 1 h of culture [175]. Hydrogels supplemented with DPSCs have shown great promise in many studies; similar to above, Luo et al. used DPSCs/heparin-poloxamer hydrogel combinations to promote viable tissue regeneration [176].

Peptide based strategies developed by the D'Souza, Hartgerink, and Kumar groups have exploited bioactive domains such as cell adhesion motifs, matrix metalloproteinase cleavable sites, heparin binding sequences and dentinogenic domains to regenerate pulp-like tissue [45,[177], [178], [179], [180], [181], [182], [183], [184], [185], [186], [187], [188], [189], [190], [191], [192], [193]]. These strategies employ short peptides of 5–50 amino acid residues which self-assemble into thixotropic hydrogels that can be syringe-aspirated and injected with 18–20 gauge needles in situ [194].

In one example, the Kumar group demonstrated *in vitro* efficacy of a dentinogenic self-assembling peptide hydrogel (SAPH) termed SLd which contains a bioactive mimic of matrix extracellular phosphoglycoprotein (MEPE) previously shown to play a vital role in dental pulp stem cell (DPSC) proliferation [45]. The C-terminal bioactive domain is adjacent to a designed self-assembling domain, and contains six repeats of alternating hydrophobic leucine and hydrophilic serine residues with flanking positively charged lysines. This unique design gives rise to spontaneously self-assembling nanofibrous beta-sheets which form a stiff hydrogel at 40 mg/mL in aqueous solution. The resulting thixotropic gel SLd showed good cytocompatibility, supported proliferation and increased calcium phosphate deposition in a dose-dependent manner. While SLd displayed great efficacy *in vitro*, it did not demonstrate comparable results *in vivo* [45]. The Kumar group has further explored the use of this dentinogenic peptide hydrogel SLd and another angiogenic peptide hydrogel, SLan, in a 1-month canine pulpectomy model. Interestingly, the carrier control and SLd induced the formation of disorganized tissue within the root canal space, while SLan caused rapid infiltration of cells extending from the apex to the crown and regenerated organized vascularized pulp-like tissue.

### 5 Outlook

The degradation of mineralized and organic tooth tissue due to poor oral hygiene results in pain and eventual loss of permanent structures within the tooth, and often requires surgical procedures to replace the infected pulp with inert materials such as gutta-percha. Recently, several strategies, including stem cell-based and cell-homing methods, have been explored to circumvent these root canal procedures to opt instead for dental pulp regeneration. Some advantages of these strategies are sufficient biocompatibility and proliferation, however, the requisite time scale (typically months-long procedures) hampers their viability in clinical settings. As an alternative, traditional materials-based strategies have been expanded upon and explored for revascularize and regeneration of hard and soft dental tissues. Traditionally, these materials are inert or are prepared as composite materials, the latter allowing for tunability though complicating validation and preparation. Synthetic materials and biomimetic materials are advantageous as revascularization of the dental pulp is achieved through growth factors or the innate ability of unique polymers to regenerate dental pulp, many of which can be harvested directly from low-cost sources, or derived directly from the patient to facilitate personalized treatment options. Materials such as peptide hydrogels confer many of the desirable physical and biological properties found in the more common regenerative endodontic materials, without the complications in validation and preparation that arise from composite materials. Recently developed angiogenic peptide hydrogels can be syringe injected and re-assemble to fill the dental cavity, simplifying their practical use and formulation, and have shown efficacy in a 1-month canine pulpectomy model. While there are still significant challenges remaining in the field of regenerative endodontics, such as long-term efficacy, new biomimetic materials-based strategies have shown promise in regenerating dental pulp.

### Author contributions

Zain Siddiqui: Writing original draft; Writing – review and editing. Amanda M. Acevedo-Jake: Writing original draft; Writing – review and editing; Figure drafting, guidance and preparation; Supervision and conceptualization; Funding acquisition. Alexandra Griffith: Writing original draft; Writing – review and editing. Nurten Kadincesme: Writing original draft; Writing – review and editing. Kinga Dabek: Writing original draft; Figure preparation. Dana Hindi: Writing original draft; Writing – review and editing. Ka Kyung Kim: Writing original draft. Yoshifumi Kobayashi: Writing – review and editing. Emi Shimizu: Writing – review and editing; Funding acquisition. Vivek Kumar: Conceptualization and supervision; Funding acquisition; Writing – review and editing; guidance with figure preparation.

Conflict of Interest Statement for Cells and Material-Based Strategies for Regenerative Endodontics.

## Declaration of competing interest

V. A. K. (corresponding author) has equity interests in start-up companies attempting to translate peptides bearing angiogenic sequences. The remaining authors declare no conflicts of interest.
